# 1291. Malignant Otitis Externa With Facial Nerve Paralysis : Comparative Analysis

**DOI:** 10.1093/ofid/ofad500.1130

**Published:** 2023-11-27

**Authors:** Fatma Hammami, Makram Koubaa, Khaoula Rekik, Amal Chakroun, Fatma Smaoui, Chakib Marrakchi, Mounir Ben Jemaa

**Affiliations:** Infectious Diseases Department, Hedi Chaker University Hospital, University of Sfax, Tunisia, Sfax, Sfax, Tunisia; Infectious Diseases Department, Hedi Chaker University Hospital, University of Sfax, Tunisia, Sfax, Sfax, Tunisia; Infectious Diseases Department, Hedi Chaker University Hospital, University of Sfax, Tunisia, Sfax, Sfax, Tunisia; Infectious Diseases Department, Hedi Chaker University Hospital, University of Sfax, Tunisia, Sfax, Sfax, Tunisia; Infectious Diseases Department, Hedi Chaker University Hospital, University of Sfax, Tunisia, Sfax, Sfax, Tunisia; Infectious Diseases Department, Hedi Chaker University Hospital, University of Sfax, Tunisia, Sfax, Sfax, Tunisia; Infectious Diseases Department, Hedi Chaker University Hospital, University of Sfax, Tunisia, Sfax, Sfax, Tunisia

## Abstract

**Background:**

Malignant otitis externa (MOE) is a life-threatening invasive infection of the external auditory canal with varying outcomes. Facial nerve paralysis (FP) is usually a sign of progression of the disease. We aimed to compare the characteristics and prognosis of patients with MOE with and without FP.

**Methods:**

We conducted a retrospective study including all patients hospitalized for MOE in the infectious diseases department between 2000 and 2022.

**Results:**

We encountered 114 cases among which 17 had a FP (14.9%). Median C-reactive protein levels (36[30-81] mg/L vs 14[7-37] mg/L ; p=0.002) and white blood cells count (9600[7800-11300] /mm^3^ vs 8270[7037-10070] /mm^3^ ; p=0.04) were significantly higher among patients with FP. Otalgia (94.1% vs 87.6% ; p=0.688), otorrhea (64.7% vs 71.1% ; p=0.593) and cephalalgia (47.1% vs 49.5% ; p=0.854) were the revealing symptoms among patients with or without FP, with no significant difference. Otoscopic examination revealed the presence of polyp in the external auditory canal more frequently among patients with FP (47.1% vs 21.6% ; p=0.036). Bone osteolysis was significantly more frequent among patients with FP (88.2% vs 54.2% ; p=0.008). Fungal species were isolated more frequently among cases with FP (70.6% vs 40.2% ; p=0.020). Complications were significantly more frequent among patients with FP (64.7% vs 26.8% ; p=0.002). Favorable evolution of the disease was more frequent among patients without FP (87.2% vs 62.5% ; p=0.024).

**Conclusion:**

MOE with FP, caused more often by fungal species, were characterised by higher inflammatory markers, the presence of polyp in otoscopic examination and bone osteolysis on imaging results. FP worsened the prognosis of the disease. Prompt and aggressive diagnosis and management are crucial.

**Disclosures:**

**All Authors**: No reported disclosures

